# Plasma Biomarkers, Hippocampal Volume and Cognitive Effects of Oral ALZ‐801: The Phase 2 Biomarker Study and its Long‐Term Extension in APOE4 Carriers with Early Alzheimer’s Disease

**DOI:** 10.1002/alz.092548

**Published:** 2025-01-09

**Authors:** Susan Abushakra, John A. Hey, Kaj Blennow, Jakub Hort, Niels D. Prins, Patrick Kesslak, Winnie Pak, Larry Shen, Xinyi Zhu, Caiyan Li, Adem Albayrak, Luc Bracoud, Joyce Suhy, Aidan Power, Martin Tolar

**Affiliations:** ^1^ 378 Santana Row, Framingham, MA USA; ^2^ Alzheon, Inc., Framingham, MA USA; ^3^ University of Gothenburg, Gothenburg Sweden; ^4^ Charles University, Second Faculty of Medicine and Motol University Hospital, Prague Czech Republic; ^5^ Brain Research Center, Amsterdam Netherlands; ^6^ Alzheon Inc., Framingham, MA USA; ^7^ Alzheon, Framingham, MA USA; ^8^ Biometrics at Pharmapace, San Diego, CA USA; ^9^ Pharmapace, San Diego, CA USA; ^10^ Clario, Inc., Lyon France; ^11^ Clario, Inc., San Mateo, CA USA

## Abstract

**Background:**

ALZ‐801 (valiltramiprosate) is an oral inhibitor of amyloid oligomer formation in development as a disease‐modifying AD treatment, including a fully enrolled APOLLOE4 Phase 3 trial in 325 APOE4/4 homozygotes. A Phase 2 study is evaluating ALZ‐801 effects on plasma biomarkers, brain volumes and cognitive outcomes in APOE4 carriers. Plasma p‐tau_181_ reduction over 104 weeks is primary endpoint. A study extension includes active treatment over additional 104 weeks for a total of 208 weeks (4 years). Long term analyses include plasma p‐tau_181_, Aβ42/40, p‐tau_217_, GFAP and other emerging biomarkers. Hippocampal volume and cognition are additional outcomes.

**Method:**

This active study enrolled 84 APOE4 carriers (MMSE ≥22; positive CSF+ biomarkers) who receive ALZ‐801 265 mg BID. CSF/plasma biomarkers were conducted at Dr. Blennow’s laboratory (Simoa, EuroImmun assays). MRIs analyzed by Clario at baseline, 52, 104, and in the long‐term extension (LTE); cognitive and biomarker tests are every 26 weeks. Biomarkers are analyzed on observed cases, hippocampal volume (HV) and cognitive tests are analyzed by MMRM with two‐sided p‐values; with Spearman’s correlations of biomarker‐clinical outcomes.

**Result:**

Baseline mean age was 69 years, MMSE 26, 51% females, and 70% MCI; and 83 subjects had any post‐treatment efficacy assessment. Total of 75 and 70 subjects completed 52 and 104 weeks. At 104 weeks, plasma p‐tau_181_ reduction was 31% (p=0.045), Aβ42 reduction was 4% (p=0.042). Hippocampal volume (HV) versus untreated matched subjects from ADNI showed ∼20% less atrophy (p<0.002). Immediate and delayed memory scores (Rey test) remained at baseline levels versus 21% decline in matched ADNI subjects (p<0.0001). Memory stabilization correlated with reduced HV atrophy (r= 0.44, p=0.0002). In the 66 ongoing LTE subjects, cognitive and HV ADNI comparisons and biomarker‐cognitive correlations at 130 weeks will be reported. Most common TEAE was mild nausea, with no ARIA‐E.

**Conclusion:**

Biomarker positive APOE4 carriers showed significant plasma p‐tau_181_ and Aβ42 effects, with promising HV effects that correlated with cognitive benefit. Sustained long term benefits on these outcomes over 2.5 years could support disease modification with a continued favorable safety profile of ALZ‐801. Correlations of plasma biomarkers to long‐term clinical and imaging outcomes provides valuable insights into ALZ‐801 effects in AD.

